# Supplementary material for: Home settings are associated with less functional decline among older adults compared to community-care foster homes and skilled nursing facilities in Hawaii
Source: PLoS One. 2025 Jun 23;20(6):e0326944. doi: 10.1371/journal.pone.0326944 (PMC12185003; doi:10.1371/journal.pone.0326944)
Supplement: S1 Fig — (DOCX) [file pone.0326944.s001.docx]

S1 Fig. Sample selection process


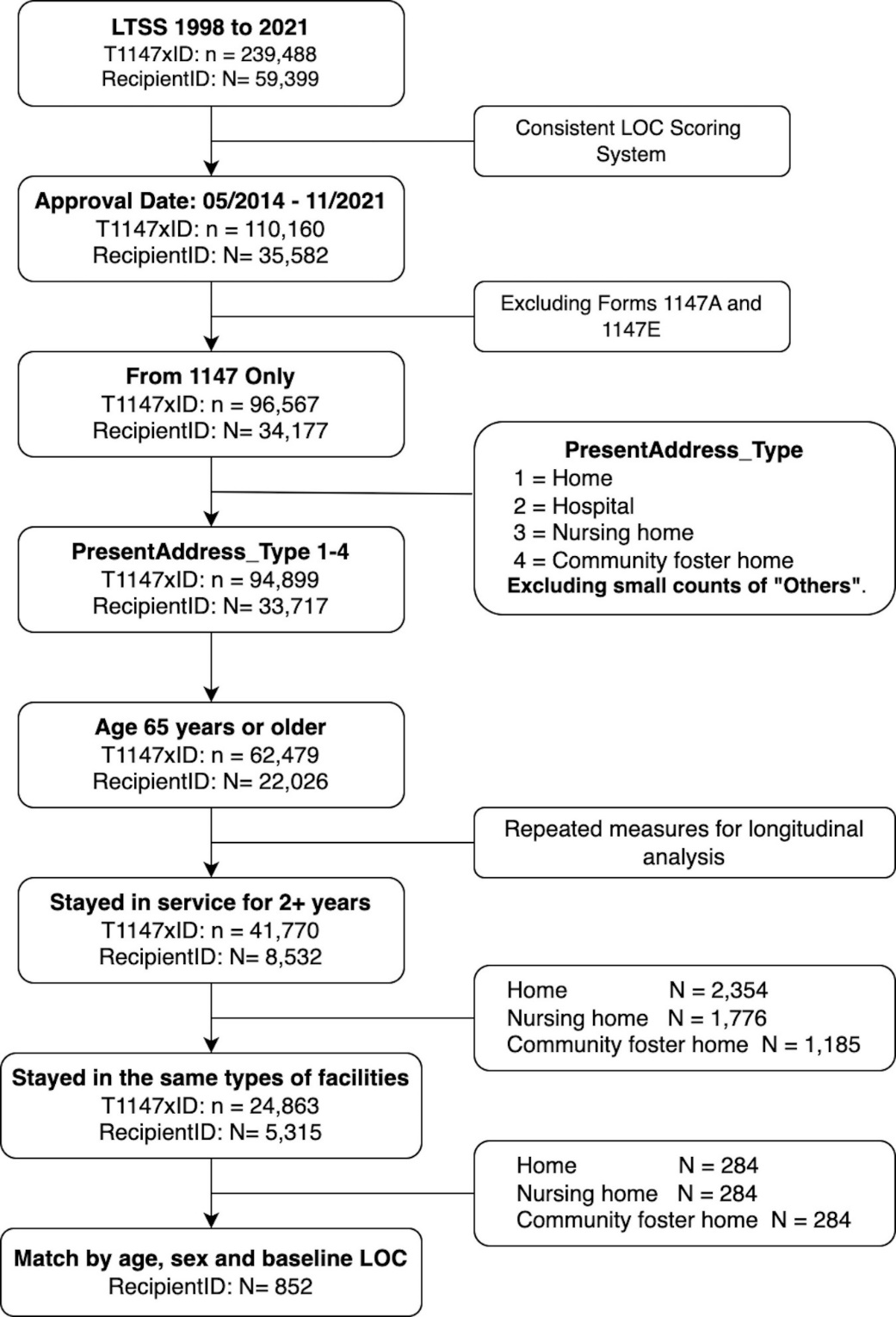


Notes: T1147xID denotes the number of level of care (LOC) assessments. RecipientID denotes the number of Medicaid beneficiaries. We restricted to years from 2014 to 2021 because there is no change in the context and structure of the assessment form during this period. We also excluded data from the short assessment form which does not have the LOC scores ([Form 1147A](https://medquest.hawaii.gov/content/dam/formsanddocuments/provider-forms/1147a-level-of-care-re-evaluation/DHS_1147A_Form_Rev_06_2023-PRINT.pdf)) and the assessment form for children/youth under 21 years old ([Form 1147E](https://medquest.hawaii.gov/content/dam/formsanddocuments/provider-forms/1147e-youth-under-age-21-level-of-care-evaluation/DHS_1147E_Form_Rev_06_2023-PRINT.pdf)). We used “foster home” and “community foster home” interchangeably in this study.
