# Supplementary material for: Home settings are associated with less functional decline among older adults compared to community-care foster homes and skilled nursing facilities in Hawaii
Source: PLoS One. 2025 Jun 23;20(6):e0326944. doi: 10.1371/journal.pone.0326944 (PMC12185003; doi:10.1371/journal.pone.0326944)
Supplement: Attachment — Submitted filename: Response to Reviewers.docx [file pone.0326944.s004.docx]

**Responses to the reviewer:**

**We appreciate the opportunity to revise and resubmit this manuscript and thank the reviewer for the insightful comments. We have revised the manuscript according to reviewers' constructive feedback. Our specific responses to each comment are below.**

**Comments to the Author**

1. If the authors have adequately addressed your comments raised in a previous round of review and you feel that this manuscript is now acceptable for publication, you may indicate that here to bypass the “Comments to the Author” section, enter your conflict of interest statement in the “Confidential to Editor” section, and submit your "Accept" recommendation.

Reviewer #1: All comments have been addressed

2. Is the manuscript technically sound, and do the data support the conclusions?

The manuscript must describe a technically sound piece of scientific research with data that supports the conclusions. Experiments must have been conducted rigorously, with appropriate controls, replication, and sample sizes. The conclusions must be drawn appropriately based on the data presented.

Reviewer #1: Partly

**RESPONSE:**

**We have revised the abstract and conclusion sections to ensure alignment between the data and the conclusions.**

3. Has the statistical analysis been performed appropriately and rigorously?

Reviewer #1: Yes

4. Have the authors made all data underlying the findings in their manuscript fully available?

The [PLOS Data policy](https://urldefense.com/v3/__http:/track.editorialmanager.com/CL0/http:*2F*2Fwww.plosone.org*2Fstatic*2Fpolicies.action*23sharing/1/010f0194260c80aa-42eb4354-e6ce-421f-b74c-7334960abae7-000000/c9IBzZy7YlrtqWFIxR7whx1M-dAiWyMvB8n9qDRwzyA=192__;JSUlJSU!!PvDODwlR4mBZyAb0!RNVSrB6SXztCDZINCB8x7V2idRDejWvNAPzQXm5Qeaglwn2wPMuo4cRDa5yMX0SQSw9SzVIRiZFy988$) requires authors to make all data underlying the findings described in their manuscript fully available without restriction, with rare exception (please refer to the Data Availability Statement in the manuscript PDF file). The data should be provided as part of the manuscript or its supporting information, or deposited to a public repository. For example, in addition to summary statistics, the data points behind means, medians and variance measures should be available. If there are restrictions on publicly sharing data—e.g. participant privacy or use of data from a third party—those must be specified.

Reviewer #1: Yes

5. Is the manuscript presented in an intelligible fashion and written in standard English?

PLOS ONE does not copyedit accepted manuscripts, so the language in submitted articles must be clear, correct, and unambiguous. Any typographical or grammatical errors should be corrected at revision, so please note any specific errors here.

Reviewer #1: Yes

6. Review Comments to the Author

Please use the space provided to explain your answers to the questions above. You may also include additional comments for the author, including concerns about dual publication, research ethics, or publication ethics. (Please upload your review as an attachment if it exceeds 20,000 characters)

Reviewer #1: The article has been substantially revised, incorporating differentiated analyses on cognition and functional status. The main finding is that residents in foster homes exhibited a greater functional decline compared to those residing in private homes.

I maintain that the conclusions and interpretations of the results should be approached with greater nuance, particularly regarding the statement: “These findings suggest that, when possible, Medicaid agencies should prioritize supporting long-term care in private homes.” Placement in nursing homes or foster homes occurs when remaining at home is either undesirable or unfeasible.

Comorbidities such as diabetes, renal insufficiency, stroke history, hemiplegia, cancer, etc., which may explain placement in nursing or foster homes, were not accounted for. While a decline in functional status is indeed observed, other parameters are equally important, including mortality, hospitalizations, quality of life, social isolation etc. These factors play a critical role in guiding policymakers and families in choosing among various housing options. It is therefore, in my opinion, inappropriate to contrast these alternatives based solely on functional status.

**RESPONSE:**

**We have revised the abstract and conclusion sections, highlighting the study's focus on functional status in the conclusion. Further research is needed to explore other health outcomes, such as mortality, hospitalization, and quality of life across care settings, to better inform effective care options for improving health.**

7. PLOS authors have the option to publish the peer review history of their article ([what does this mean?](https://urldefense.com/v3/__https:/track.editorialmanager.com/CL0/https:*2F*2Fjournals.plos.org*2Fplosone*2Fs*2Feditorial-and-peer-review-process*23loc-peer-review-history/1/010f0194260c80aa-42eb4354-e6ce-421f-b74c-7334960abae7-000000/Pd6-ybG20-tDYPG23M6BUnIuc0PUYMIb5Uw01n0xX-c=192__;JSUlJSUl!!PvDODwlR4mBZyAb0!RNVSrB6SXztCDZINCB8x7V2idRDejWvNAPzQXm5Qeaglwn2wPMuo4cRDa5yMX0SQSw9SzVIR0VoJcCE$)). If published, this will include your full peer review and any attached files.


If you choose “no”, your identity will remain anonymous but your review may still be made public.


**Do you want your identity to be public for this peer review?** For information about this choice, including consent withdrawal, please see our [Privacy Policy](https://urldefense.com/v3/__https:/track.editorialmanager.com/CL0/https:*2F*2Fwww.plos.org*2Fprivacy-policy/1/010f0194260c80aa-42eb4354-e6ce-421f-b74c-7334960abae7-000000/CWaBMdRopNt6BGmeUMQKPgMHbDXAbCB6Qi2YoET33fk=192__;JSUl!!PvDODwlR4mBZyAb0!RNVSrB6SXztCDZINCB8x7V2idRDejWvNAPzQXm5Qeaglwn2wPMuo4cRDa5yMX0SQSw9SzVIRyz_5AP4$).

Reviewer #1: **Yes:** BOUCAUD-MAITRE
