# Supplementary material for: Home settings are associated with less functional decline among older adults compared to community-care foster homes and skilled nursing facilities in Hawaii
Source: PLoS One. 2025 Jun 23;20(6):e0326944. doi: 10.1371/journal.pone.0326944 (PMC12185003; doi:10.1371/journal.pone.0326944)
Supplement: Attachment — Submitted filename: Response to Reviewers comments 5-12-25.docx [file pone.0326944.s005.docx]

**Responses to the editor and the reviewer:**

**We appreciate the opportunity to revise and resubmit this manuscript and thank you for the insightful comments. We have revised the manuscript according to the constructive feedback. Our specific responses to each comment are below.**

PONE-D-24-22552R2

Home settings are associated with less functional decline among older adults compared to community-care foster homes and skilled nursing facilities in Hawaii

PLOS ONE

Dear Dr. Zan,

Thank you for submitting your manuscript to PLOS ONE. After careful consideration, we feel that it has merit but does not fully meet PLOS ONE’s publication criteria as it currently stands. Therefore, we invite you to submit a revised version of the manuscript that addresses the points raised during the review process.

1. Overall, the clarity of certain sections of the manuscript, particularly the Methods section, require improvement to enhance readers' understanding of the analyses performed and to ensure reproducibility. For instance, the following statement lacks clarity: “Individuals at home were matched to those living in foster homes and nursing homes respectively, and then the two separate matched samples were merged by individuals in the home setting." Specifically, the meaning of “merged by individuals in the home setting” is unclear and needs further explanation.

**RESPONSE:**

**We have revised to increase clarity of the manuscript especially the Methods section. For example, we revised “Individuals at home were matched to those living in foster homes and nursing homes respectively, and then the two separate matched samples were merged by individuals in the home setting” to “****Because the matching method can only match two groups at a time, we first matched individuals at home to those living in foster homes and nursing homes respectively. Then, the two separate matched samples were linked by the individuals in the home setting, ensuring that all three settings were matched.”.**

2. It is also pertinent to explicitly include the regression equations used for the analysis to provide transparency and facilitate reproducibility.

**RESPONSE:**

**Good suggestion. We added the regression equation to the manuscript.**

3. Furthermore, illustrating the estimated slopes using figures would significantly improve the presentation and understanding of results. For reference, the figures presented in the article titled “Poor hemorrhagic stroke outcomes during the COVID-19 pandemic are driven by socioeconomic disparities: analysis of nationally representative data” (BMJ Neurology Open) offer a good example of how to visualize equations involving change in slope.

**RESPONSE:**

**Thank you for highlighting the illustration method used in the article title “Poor hemorrhagic stroke outcomes during the COVID-19 pandemic are driven by socioeconomic disparities: analysis of nationally representative data” (hereafter referred as the BMJ article). Upon closer examination, we have noted that the BMJ article and our current work addressed distinct research questions, which led us to use different analytical approaches and visualization techniques.**

**The BMJ article's objective was to determine the impact of the COVID-19 pandemic on intracerebral hemorrhage mortality, effectively visualized in their Figure 1 by illustrating the change in the slope of mortality trends between the pre-pandemic and pandemic periods using logistic regression models.**

**In contrast, our study focuses on comparing functionality, as measured by Level of Care (LOC) scores, across three different settings—home, foster home, and nursing home—over time. To achieve this, we utilized linear mixed-effects (LME) models. Our primary goal is to estimate the average baseline LOC scores for each setting (represented by the fixed-effects intercepts in our LME models) and to quantify the change in LOC scores over time within each setting (captured by the fixed-effect slopes). We believe that Figure 1 in our manuscript effectively illustrates these key findings.**

4. Additionally, clarification is required for the following statement: “In addition, since the assessment form covers various aspects of LOC needs—such as ADL, which are comparable to functional status scales in the literature, and cognition—5 we further compared the average scores for each assessment item by setting after matching and analyzed the change in LOC scores for ADL (Items VII-XIII in Form 1147) and cognition (Items V-VI) separately by setting.” The phrasing is presently ambiguous, particularly regarding the comparison and analysis of LOC scores. A more precise description would greatly enhance the reader’s comprehension.

**RESPONSE:**

**We revised and changed it to the following:**

**“The assessment form covers various aspects of LOC needs such as communication, memory, mobility, and dressing and grooming. To determine how these needs vary across settings, we compared the average scores for each assessment item by setting after matching. Given the established link between cognitive decline and increased care needs and worsened health outcomes (Connell et al., 2001; Millán-Calenti et al., 2013), we further aggregated cognition items (Items V-VI in Form 1147) and ADL items (Items VII-XIII in Form 1147). By comparing the scores of these two broad categories of care needs across settings, we aim to determine the influence of the change in cognition and functional status on shifts in LOC scores within the three settings.”**

**Reference:**

**Connell, C. M., Janevic, M. R., & Gallant, M. P. (2001). The costs of caring: impact of dementia on family caregivers. *Journal of geriatric psychiatry and neurology*, *14*(4), 179-187.**

**Millán-Calenti, J. C., Tubío, J., Pita-Fernández, S., Rochette, S., Lorenzo, T., & Maseda, A. (2012). Cognitive impairment as predictor of functional dependence in an elderly sample. *Archives of gerontology and geriatrics*, *54*(1), 197-201.**

5. While the manuscript attempts to justify why race and LOC remained statistically significant despite matching, such results often point to imperfect matching and residual confounding, which could bias the conclusions. To address this, I strongly recommend evaluating and reporting balance metrics for the propensity score model, utilizing tools such as standardized mean differences or visual plots to assess covariate balance. Additionally, exploring alternative methods, including propensity score stratification or inverse probability weighting (IPW), may help to address the issue of imperfect balance and improve the robustness of the findings.

**RESPONSE:**

**Thank you for the suggestion. To ensure an adequate sample size and maximize statistical power for detecting group differences, we employed various strategies and implemented stringent matching criteria. While the matching was not perfect, we included the matching variables as covariates in the regression models to mitigate the impact of any residual imbalance and enhance the robustness of our findings. We also acknowledged this limitation regarding imperfect balance in the discussion section.**

6. Finally, it is concerning that the authors evaluated the Medicaid population aged 65 and older without addressing the issue of dual eligibles or discussing how their dual status might influence the results.

It is equally important to provide context on how these older adults came to be Medicaid recipients. Such omissions leave critical gaps in the interpretation of the findings and the broader implications of the study. I strongly recommend that the authors include a discussion of dual eligibility and its potential impact, as well as a clear explanation of the circumstances leading to older adults being enrolled in Medicaid.

**RESPONSE:**

**Thank you for these comments. Because our sample was limited to Medicaid members over 65, they are all eligible for Medicare, all receiving Medicaid, and thus are all dually eligible. Since all participants are dually eligible, this would not affect the results of our analysis, which aimed to determine how delivery of HCBS services, specifically living and treatment context (home, foster home, or nursing facility) impacted functional outcomes over time. However, we see your point that members' dual eligibility status is important contextual information for the introduction and discussion.**

**Dual eligibles tend to have worse health outcomes, higher social needs, higher healthcare costs, racial disparities, and have less economic resources compared to non-Medicaid receiving older adults. Furthermore, individuals within our sample face additional challenges and vulnerabilities (even compared to other dual eligibles) as they have concurrent disability (defined by functional impairment) which makes them eligible for HCBS.**

**This constellation of social and medical risk factors are exactly the complex health challenges that HCBS intend to address, and framing within this social economic context is important. As for how individuals become eligible for Medicaid, we have included further details on eligibility for Medicaid. We added:**

**“To qualify for Medicaid in Hawaii, applicants must be state residents, meet specific citizenship or immigration requirements, and demonstrate limited financial resources. Income and asset limitations vary depending on the specific Medicaid program. In addition, accessing long-term care services and supports, such as HCBS and nursing home care, requires an assessment to confirm the functional need for such care. Upon approval following this evaluation, individuals gain access to tailored services and supports.”**

**In summary, we have added more discussion of challenges common to dual eligibles, added dual eligible as a keyword, and framed these findings as contributing additionally to research on services for dual eligibles receiving HCBS. Thank you for bringing this to our attention.**

Please submit your revised manuscript by May 14 2025 11:59PM. If you will need more time than this to complete your revisions, please reply to this message or contact the journal office at [plosone@plos.org](mailto:plosone@plos.org). When you're ready to submit your revision, log on to [https://www.editorialmanager.com/pone/](https://urldefense.com/v3/__https:/www.editorialmanager.com/pone/__;!!PvDODwlR4mBZyAb0!RnauSbwWtYjDkjUIcuS0IDhWKRi50VOuJjjumFW86TOnUP8_ARUxbz43DcY4C6XnjeIObfmutoa7Lok$) and select the 'Submissions Needing Revision' folder to locate your manuscript file.

Please include the following items when submitting your revised manuscript:

- A rebuttal letter that responds to each point raised by the academic editor and reviewer(s). You should upload this letter as a separate file labeled 'Response to Reviewers'.
- A marked-up copy of your manuscript that highlights changes made to the original version. You should upload this as a separate file labeled 'Revised Manuscript with Track Changes'.
- An unmarked version of your revised paper without tracked changes. You should upload this as a separate file labeled 'Manuscript'.

If you would like to make changes to your financial disclosure, please include your updated statement in your cover letter. Guidelines for resubmitting your figure files are available below the reviewer comments at the end of this letter.

If applicable, we recommend that you deposit your laboratory protocols in [protocols.io](http://protocols.io/) to enhance the reproducibility of your results. Protocols.io assigns your protocol its own identifier (DOI) so that it can be cited independently in the future. For instructions see: [https://journals.plos.org/plosone/s/submission-guidelines#loc-laboratory-protocols](https://urldefense.com/v3/__https:/track.editorialmanager.com/CL0/https:*2F*2Fjournals.plos.org*2Fplosone*2Fs*2Fsubmission-guidelines*23loc-laboratory-protocols/1/010f0195ea08822f-a8eb6ebb-3abd-4f12-8dd3-a8fa5a9d1859-000000/uQKK_N_yglbP3Wz6FnLUVjTv0W7GytBDeyqxlYjmUhA=204__;JSUlJSUl!!PvDODwlR4mBZyAb0!RnauSbwWtYjDkjUIcuS0IDhWKRi50VOuJjjumFW86TOnUP8_ARUxbz43DcY4C6XnjeIObfmumGwMgrM$). Additionally, PLOS ONE offers an option for publishing peer-reviewed Lab Protocol articles, which describe protocols hosted on [protocols.io](http://protocols.io/). Read more information on sharing protocols at [https://plos.org/protocols?utm_medium=editorial-email&utm_source=authorletters&utm_campaign=protocols](https://urldefense.com/v3/__https:/track.editorialmanager.com/CL0/https:*2F*2Fplos.org*2Fprotocols*3Futm_medium=editorial-email*26utm_source=authorletters*26utm_campaign=protocols/1/010f0195ea08822f-a8eb6ebb-3abd-4f12-8dd3-a8fa5a9d1859-000000/vWTh4iT38H2GQ-ymsZpeGTG2jybE3dIeeZ_hipAB678=204__;JSUlJSUl!!PvDODwlR4mBZyAb0!RnauSbwWtYjDkjUIcuS0IDhWKRi50VOuJjjumFW86TOnUP8_ARUxbz43DcY4C6XnjeIObfmucgXASzo$).

We look forward to receiving your revised manuscript.

Kind regards,

Abdulaziz T. Bako, MBBS; MPH; PhD
Academic Editor
PLOS ONE

Journal Requirements:

Please review your reference list to ensure that it is complete and correct. If you have cited papers that have been retracted, please include the rationale for doing so in the manuscript text, or remove these references and replace them with relevant current references. Any changes to the reference list should be mentioned in the rebuttal letter that accompanies your revised manuscript. If you need to cite a retracted article, indicate the article’s retracted status in the References list and also include a citation and full reference for the retraction notice.


[Note: HTML markup is below. Please do not edit.]

Reviewers' comments:

Reviewer's Responses to Questions

**Comments to the Author**

1. If the authors have adequately addressed your comments raised in a previous round of review and you feel that this manuscript is now acceptable for publication, you may indicate that here to bypass the “Comments to the Author” section, enter your conflict of interest statement in the “Confidential to Editor” section, and submit your "Accept" recommendation.

Reviewer #1: All comments have been addressed

2. Is the manuscript technically sound, and do the data support the conclusions?

The manuscript must describe a technically sound piece of scientific research with data that supports the conclusions. Experiments must have been conducted rigorously, with appropriate controls, replication, and sample sizes. The conclusions must be drawn appropriately based on the data presented.

Reviewer #1: Yes

3. Has the statistical analysis been performed appropriately and rigorously?

Reviewer #1: Yes

4. Have the authors made all data underlying the findings in their manuscript fully available?

The [PLOS Data policy](https://urldefense.com/v3/__http:/track.editorialmanager.com/CL0/http:*2F*2Fwww.plosone.org*2Fstatic*2Fpolicies.action*23sharing/1/010f0195ea08822f-a8eb6ebb-3abd-4f12-8dd3-a8fa5a9d1859-000000/QzuwbdWJT0O4pFSHJeVXrqMHJuPL9ktgo5yarE0T43A=204__;JSUlJSU!!PvDODwlR4mBZyAb0!RnauSbwWtYjDkjUIcuS0IDhWKRi50VOuJjjumFW86TOnUP8_ARUxbz43DcY4C6XnjeIObfmua-kbOEU$) requires authors to make all data underlying the findings described in their manuscript fully available without restriction, with rare exception (please refer to the Data Availability Statement in the manuscript PDF file). The data should be provided as part of the manuscript or its supporting information, or deposited to a public repository. For example, in addition to summary statistics, the data points behind means, medians and variance measures should be available. If there are restrictions on publicly sharing data—e.g. participant privacy or use of data from a third party—those must be specified.

Reviewer #1: Yes

5. Is the manuscript presented in an intelligible fashion and written in standard English?

PLOS ONE does not copyedit accepted manuscripts, so the language in submitted articles must be clear, correct, and unambiguous. Any typographical or grammatical errors should be corrected at revision, so please note any specific errors here.

Reviewer #1: Yes

6. Review Comments to the Author

Please use the space provided to explain your answers to the questions above. You may also include additional comments for the author, including concerns about dual publication, research ethics, or publication ethics. (Please upload your review as an attachment if it exceeds 20,000 characters)

Reviewer #1: Dear authors,

thank you for taking into account my latest comments, which help to qualify your results, and congratulations on your article.

7. PLOS authors have the option to publish the peer review history of their article ([what does this mean?](https://urldefense.com/v3/__https:/track.editorialmanager.com/CL0/https:*2F*2Fjournals.plos.org*2Fplosone*2Fs*2Feditorial-and-peer-review-process*23loc-peer-review-history/1/010f0195ea08822f-a8eb6ebb-3abd-4f12-8dd3-a8fa5a9d1859-000000/ZxGpohbcbXCOsRN3v0bkOGYq5nJtHGnMezqdv-Hw8b0=204__;JSUlJSUl!!PvDODwlR4mBZyAb0!RnauSbwWtYjDkjUIcuS0IDhWKRi50VOuJjjumFW86TOnUP8_ARUxbz43DcY4C6XnjeIObfmumjItT2Y$)). If published, this will include your full peer review and any attached files.


If you choose “no”, your identity will remain anonymous but your review may still be made public.


**Do you want your identity to be public for this peer review?** For information about this choice, including consent withdrawal, please see our [Privacy Policy](https://urldefense.com/v3/__https:/track.editorialmanager.com/CL0/https:*2F*2Fwww.plos.org*2Fprivacy-policy/1/010f0195ea08822f-a8eb6ebb-3abd-4f12-8dd3-a8fa5a9d1859-000000/RhXCsAgGva2AbykwE1z7n58jrq-0GuSBPt402CtdO-M=204__;JSUl!!PvDODwlR4mBZyAb0!RnauSbwWtYjDkjUIcuS0IDhWKRi50VOuJjjumFW86TOnUP8_ARUxbz43DcY4C6XnjeIObfmu8mAwhMs$).

Reviewer #1: **Yes:** Boucaud-Maitre

[NOTE: If reviewer comments were submitted as an attachment file, they will be attached to this email and accessible via the submission site. Please log into your account, locate the manuscript record, and check for the action link "View Attachments". If this link does not appear, there are no attachment files.]

While revising your submission, please upload your figure files to the Preflight Analysis and Conversion Engine (PACE) digital diagnostic tool, [https://pacev2.apexcovantage.com/](https://urldefense.com/v3/__https:/track.editorialmanager.com/CL0/https:*2F*2Fpacev2.apexcovantage.com*2F/1/010f0195ea08822f-a8eb6ebb-3abd-4f12-8dd3-a8fa5a9d1859-000000/qwKxsdXQ98bST5F8UaGv8Pg3_HjdVc7fE-vf1p_PwWg=204__;JSUl!!PvDODwlR4mBZyAb0!RnauSbwWtYjDkjUIcuS0IDhWKRi50VOuJjjumFW86TOnUP8_ARUxbz43DcY4C6XnjeIObfmug3is6cM$). PACE helps ensure that figures meet PLOS requirements. To use PACE, you must first register as a user. Registration is free. Then, login and navigate to the UPLOAD tab, where you will find detailed instructions on how to use the tool. If you encounter any issues or have any questions when using PACE, please email PLOS at [figures@plos.org](mailto:figures@plos.org). Please note that Supporting Information files do not need this step.
